# Methyl Benzoate Acts as a Dusk‐Associated Airborne Cue Activating NPR1‐Dependent Defence Signalling

**DOI:** 10.1111/pce.70754

**Published:** 2026-07-14

**Authors:** Riichiro Yoshida, Nguyen Thi Duyen, Nao Shimomai, Yuma Tanaka

**Affiliations:** ^1^ Laboratory of Horticultural Science, Faculty of Agriculture Kagoshima University Kagoshima Kagoshima Japan; ^2^ The United Graduate School of Agricultural Sciences Kagoshima University Kagoshima Kagoshima Japan

## Abstract

Methyl benzoate (MB) is emitted around dusk and functions as an airborne cue that activates NPR1‐dependent defense signaling. Our findings identify MB as a previously unrecognized volatile defense signal distinct from methyl salicylate and expand our understanding of airborne communication in plants.

Plants use volatile organic compounds (VOCs) as airborne signals to coordinate defence responses within and between individuals (Heil and Ton 2008; Erb 2018; Brosset and Blande 2022; Bergman et al. 2025). Several VOCs can prime or induce plant immunity, yet only a limited number have been functionally characterized as bona fide immune signals with defined molecular pathways. Methyl salicylate (MeSA) is the best‐characterized example, acting through of salicylic acid (SA)‐associated signalling via the master regulator *NONEXPRESSOR OF PATHOGENESIS‐RELATED GENES 1* (*NPR1*) (Park et al. 2007; Vlot et al. 2009; Fu and Dong 2013; Backer et al. 2019). Whether additional volatile esters function as airborne defence cues remains largely unexplored.

Methyl benzoate (MB) is a widespread plant volatile emitted from flowers, fruits and leaves of many species (Knudsen et al. 2006). In addition to its role in floral scent, MB has been reported to affect insect behaviour (Feng and Zhang 2017), although its role in plant defence signalling remains unclear. Here, we show that MB functions as an airborne immune signal that induces *PATHOGENESIS‐RELATED* (*PR*) gene expression through an NPR1‐dependent pathway, with responses retained in the *sid2‐2* background, revealing signalling behaviour distinct from MeSA.

To confirm MB emission from vegetative tissues, we analyzed headspace volatiles from intact tomato leaves during day and night using gas chromatography–mass spectrometry (GC–MS) (Supporting Information S1: Figure 1). A distinct MB peak was consistently detected. Time‐course quantification across a diel cycle under a 14‐h photoperiod revealed a significant temporal emission pattern, with peak MB release observed around ZT12–16 (Figure 1a). Supporting analyses across Solanaceae species indicated interspecific variation in MB emission behaviour (Supporting Information S2: Figure 2), with detectable emission in several species but differing temporal patterns. These observations suggest that MB emission is physiologically regulated and may contribute to airborne plant signalling.

**Figure 1:**
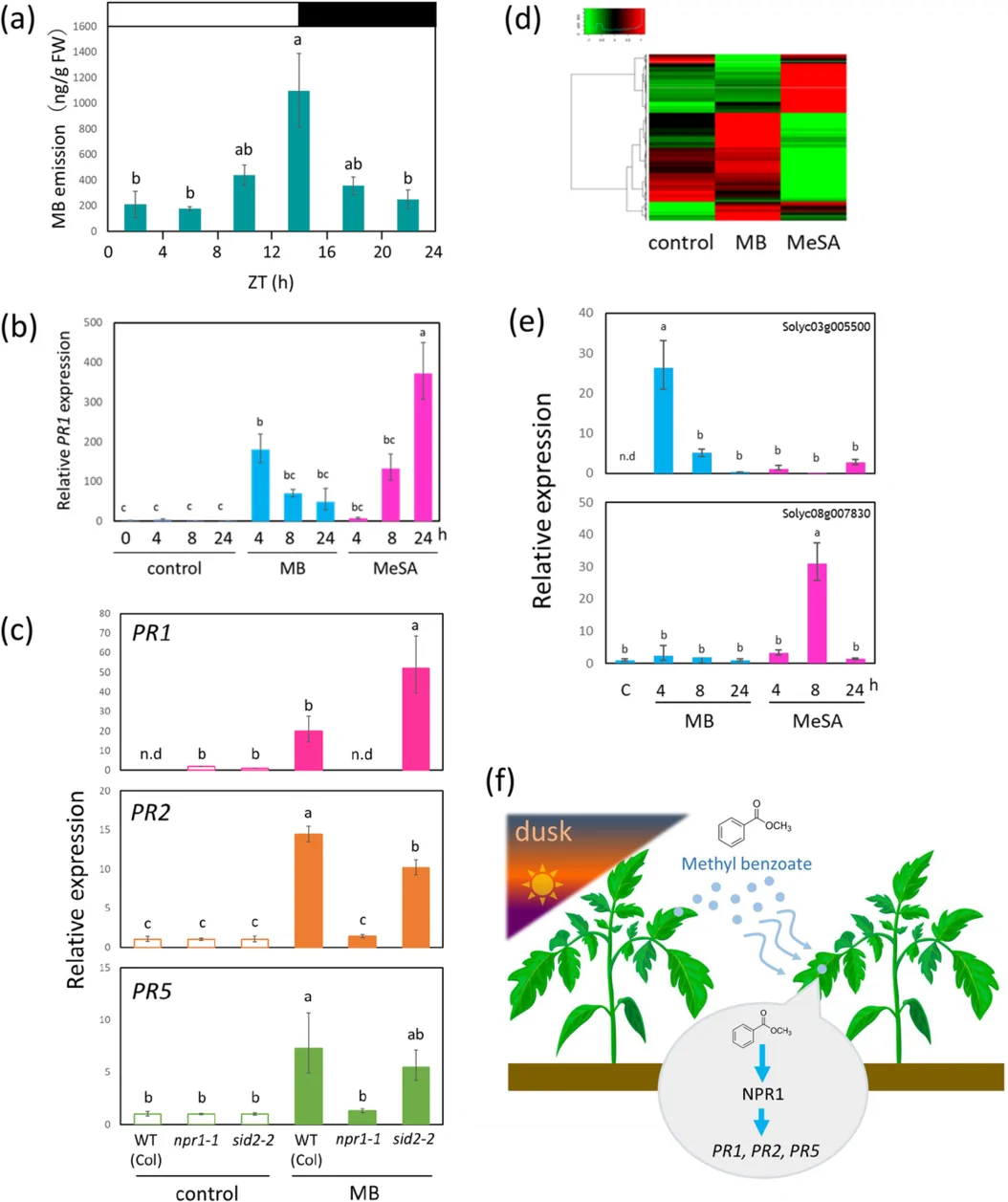
Methyl benzoate activates NPR1‐dependent defence signalling through a pathway distinct from MeSA. (a) Diurnal variation in methyl benzoate (MB) emission from intact tomato leaves under a 14‐h light/10‐h dark photoperiod. MB emission was quantified at the indicated Zeitgeber time (ZT) intervals by SPME‐GC/MS. ZT0 and ZT14 indicate lights‐on and lights‐off, respectively. Data represent mean ± SD (*n* = 3). Different letters indicate significant differences among time points (*p* < 0.05, one‐way ANOVA followed by Tukey's test). (b) *PR1* transcript levels in tomato leaves after treatment with MB (1.35 µg/cm^3^) or MeSA (1.35 µg/cm^3^) for 0, 4, 8 and 24 h. Expression levels were quantified by qRT‐PCR and normalized to *SlACT*. Data represent mean ± SD (*n* = 3). Different letters indicate significant differences among time points (*p* < 0.05, Tukey's HSD). (c) *PR1*, *PR2* and *PR5* transcript levels in wild‐type (Col‐0), *npr1‐1* and *sid2‐2 Arabidopsis* plants after MB treatment (2.7 µg/cm^3^) for 0, 4, 8 and 24 h. Expression levels were quantified by qRT‐PCR and normalized to *ACTIN2*. Data represent mean ± SD (*n* = 3). Different letters indicate significant differences among time points (*p* < 0.05, Tukey's HSD). (d) Heatmap showing differentially expressed genes identified by RNA‐seq analysis in tomato leaves treated with control, MB or MeSA. Colour intensity indicates relative expression levels. (e) Expression patterns of transcription factor genes showing differential responsiveness to MB and MeSA, based on RNA‐seq analysis. Expression levels were quantified by qRT‐PCR and normalized to *SlACT*. Data represent mean ± SD (n = 3). Different letters indicate significant differences among treatments (*p* < 0.05, Tukey's HSD). (f) Proposed model for MB‐mediated activation of NPR1‐dependent defence signalling. Temporally regulated MB emission functions as an airborne cue that activates defence gene expression through NPR1.

We next examined whether MB activates defence‐associated gene expression. Exposure of tomato plants to MB vapour rapidly induced *PR1* expression, with peak induction observed at 4 h, whereas MeSA induced a slower but progressively increasing response (Figure 1b). Additional analyses showed that MB also induced expression of *PR2 and PR5* (Figure 1c). These contrasting temporal patterns suggest that MB and MeSA engage partially distinct defence signalling mechanisms. The rapid but transient *PR1* induction triggered by MB contrasts with the slower and sustained response to MeSA, consistent with the possibility that these volatiles differ in perception, signal transduction, or metabolic processing. Similar MB responsiveness was also observed in other Solanaceae species (Supporting Information S3: Figure 3). MB‐induced transcriptional activation occurred without visible stress symptoms, supporting the interpretation that MB functions as a signalling cue rather than a general stressor. MB pretreatment also enhanced subsequent wound‐induced *PR1* expression (Supporting Information S4: Figure 4), suggesting possible priming activity.

To assess signalling requirements underlying MB responsiveness, we tested *Arabidopsis* mutants impaired in defence signalling pathways. MB‐induced *PR1* expression was strongly impaired in the *npr1‐1* mutant, indicating a requirement for NPR1 (Figure 1b). In contrast, MB responsiveness was retained in *sid2‐2*, suggesting that MB signalling differs from canonical MeSA–associated signalling.

To further characterize MB‐induced responses, we performed RNA sequencing of tomato leaves following MB or MeSA treatment. While both volatiles induced overlapping transcriptional responses, MB also activated a distinct subset of genes, including defence‐associated transcription factors such as *WRKY33*, *WRKY40* and *ERF1* (Figure 1d,e and Supporting Information S5 and S6: Figures 5 and 6). These findings support the idea that MB and MeSA trigger overlapping but distinguishable transcriptional programmes. The differential responsiveness of transcription factor genes further suggests that volatile‐specific regulatory components may contribute to shaping downstream defence outputs.

The requirement for NPR1 in MB‐induced *PR1* expression highlights its role as an important integrator of volatile‐triggered defence signals. NPR1 is well established as a central regulator of SA‐associated defence responses, transducing redox and hormonal cues into defence gene activation (Fu and Dong 2013; Backer et al. 2019). Our findings extend this framework by suggesting that NPR1 can also mediate defence activation triggered by MB, a volatile cue with response characteristics distinct from MeSA. The convergence of multiple volatile signals on NPR1 may allow plants to integrate diverse environmental chemical cues while maintaining flexibility in defence regulation. Such convergence may provide an efficient mechanism for integrating multiple airborne cues while preserving signalling specificity at earlier regulatory steps.

Based on these findings, we propose a model in which MB functions as a dusk‐associated airborne cue that activates NPR1‐dependent defence gene expression (Figure 1f). In contrast, MeSA is known to function through SA‐associated signalling prior to NPR1 activation (Park et al. 2007). These observations suggest that NPR1 may serve as a convergence point for multiple volatile defence cues while distinct upstream signalling routes shape transcriptional outputs. Taken together, our findings identify MB as a previously unrecognized airborne cue that activates NPR1‐dependent defence signalling. Unlike MeSA, MB elicits distinct transcriptional and temporal response patterns, supporting the existence of mechanistically distinguishable volatile signalling routes. Given its widespread occurrence in plants, MB may represent an additional component of volatile‐mediated plant defence communication, expanding the repertoire of airborne chemical signals linked to NPR1‐dependent defence regulation.

## Supporting information


**Figure S1**: Day–night comparison of MB emission in tomato leaves. Volatiles were collected by SPME from intact tomato plants during daytime (10:00–17:00) and nighttime (18:00–08:00) under a 14 h light/10 h dark photoperiod. Representative GC–MS chromatograms show a distinct MB peak (retention time 11.2 min; inset: mass spectrum).

**Figure S2**: Day–night comparison of MB emission among Solanaceae species. Volatile emissions were collected using SPME from intact leaves of five Solanaceae species— pepper (*Capsicum annuum*), eggplant (*Solanum melongena*), tobacco (*Nicotiana sylvestris*), potato (*Solanum tuberosum*) and *Physalis peruviana*—during daytime (10:00–17:00) and nighttime (18:00–08:00) under a 14 h light/10 h dark photoperiod. In all species except eggplant and tobacco, MB emission was higher at night than during the day. The differences were statistically significant in pepper (*p* < 0.01), potato and *Physalis* (*p* < 0.05). MB was not detected in eggplant. Bars represent mean ± SD (*n* = 5). Asterisks indicate significant differences between day and night periods (*p < 0.05, **p < 0.01, ns: not significant, *t*‐test). n.d, not detected.

**Figure S3**: Time‐course of *PR1* expression induced by MB and MeSA in Solanaceae plants. *PR1* expression in pepper and eggplant leaves following MB treatment (1.35 µg/cm^3^). Gene expression was quantified by qRT‐PCR and normalized to *Ca18S* (for pepper) and *SmACT* (for eggplant) genes. Bars represent mean ± SD (*n* = 3). Different letters indicate significant differences among time points (*p* < 0.05, Tukey's HSD).

**Figure S4**: Priming effect of MB on wound‐induced *PR1* expression in tomato leaves. Tomato plants were pre‐exposed to MB (1.35 µg/cm^3^) or control air for 24 h and subsequently subjected to mechanical wounding by making shallow incisions on leaf blades using scissors. Relative *PR1* transcript levels were quantified by qRT‐PCR at 0, 2, 5 and 10 h after wounding. MB‐treated plants exhibited a pronounced priming effect, characterized by earlier and stronger induction of *PR1* expression compared with control plants. Data represent means ± SD (*n* = 3). Different letters denote significant differences among treatments at each time point (Tukey's HSD test, *p* < 0.05).

**Figure S5**: Comparative transcriptome analysis of tomato leaves treated with MB or MeSA. (a) Volcano plot showing differentially expressed genes (DEGs) between control and MB‐treated (1.35 µg/cm^3^) leaves. (b) Volcano plot showing DEGs between control and MeSA‐treated (1.35 µg/cm^3^) leaves. Significantly upregulated and downregulated genes (|log_2_FC| ≥ 1, FDR < 0.05) are shown in red and blue (a) or orange and green (b), respectively. (c) Venn diagram illustrating the overlap and specificity of differentially expressed tomato genes induced by MB and MeSA relative to the control treatment. (d) Gene Ontology (GO) enrichment analysis of MB up‐ and down‐regulated genes. Top 15 enriched GO terms (FDR < 0.05) are shown as a bubble plot; bubble size represents gene number and colour denotes −log_10_(FDR). (e) GO enrichment analysis of MeSA up‐ and down‐regulated genes. Top 13 enriched GO terms (FDR < 0.05) are shown as a bubble plot; bubble size represents gene number and colour denotes −log_10_(FDR).

**Figure S6**: Differential expression and validation of transcription factors induced by MB and MeSA. Heatmap showing transcription factor genes differentially expressed in tomato leaves treated with MB or MeSA. Only transcription factor genes with |fold change| ≥ 3 and *p* < 0.05 were included. Colour intensity indicates *Z*‐score normalized expression values derived from RNA‐seq data.

**Table S1:** Information on primers and genes used in this study.

Supporting File 1

## Data Availability

Data sharing is not applicable to this article as no data sets were generated or analyzed during the current study.
