# Supplementary material for: Methyl Benzoate Acts as a Dusk‐Associated Airborne Cue Activating NPR1‐Dependent Defence Signalling
Source: Plant Cell Environ. 2026 Jul 14;49(11):8400–3. doi: 10.1111/pce.70754 (PMC13646652; doi:10.1111/pce.70754)
Supplement: Supplementary file 1 [file PCE-49-8400-s001.pdf]

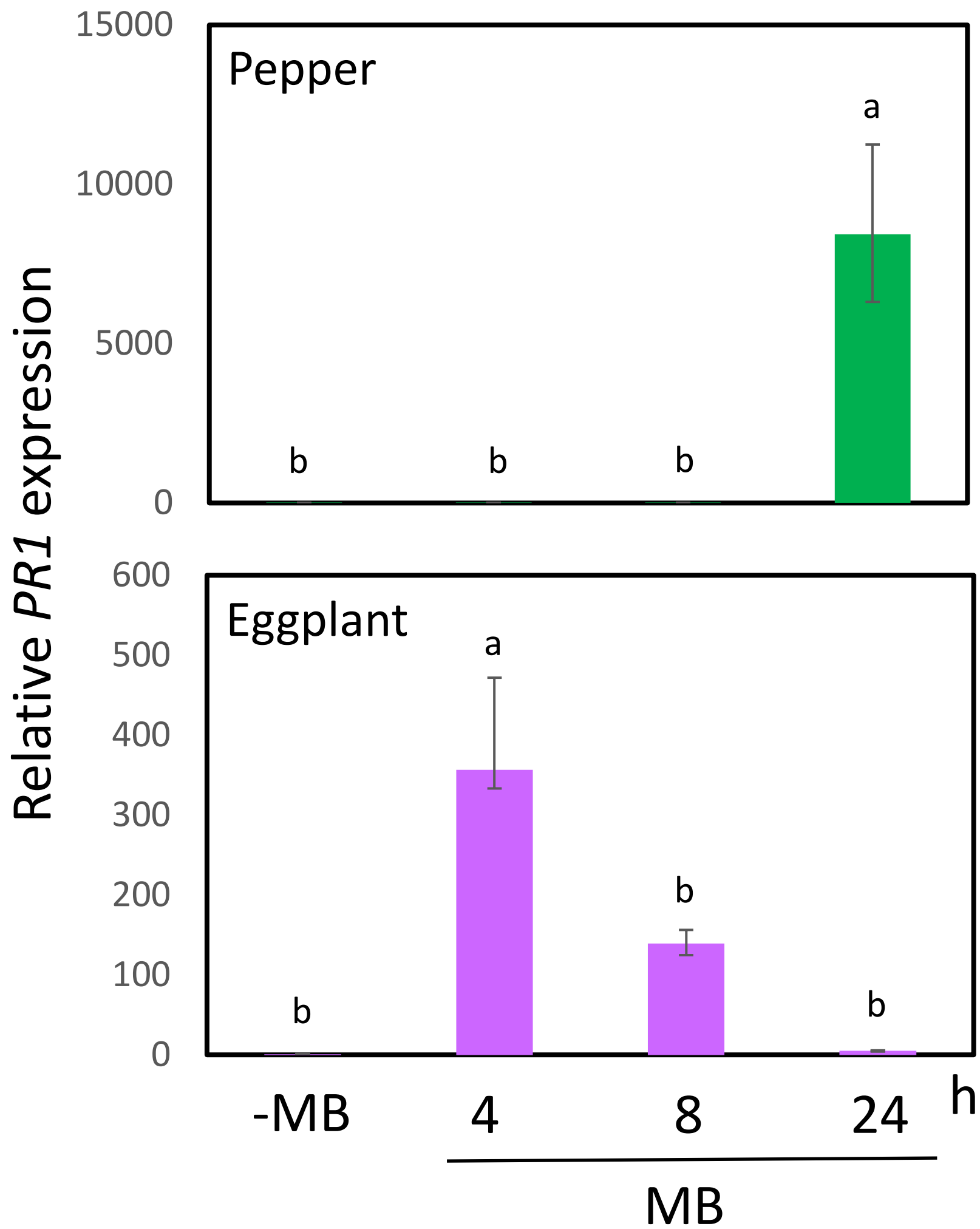

Supporting Figure S3

Time-course of *PR1* expression induced by MB and MeSA in *Solanaceae* plants.

PR1 expression in pepper and eggplant leaves following MB treatment (1.35 μg/cm^3). Gene expression was quantified by qRT–PCR and normalized to *Ca18S* (for pepper) and *SmACT* (for eggplant) genes. Bars represent mean ± SD (n = 3). Different letters indicate significant differences among time points (P < 0.05, Tukey's HSD).
