# Supplementary material for: Methyl Benzoate Acts as a Dusk‐Associated Airborne Cue Activating NPR1‐Dependent Defence Signalling
Source: Plant Cell Environ. 2026 Jul 14;49(11):8400–3. doi: 10.1111/pce.70754 (PMC13646652; doi:10.1111/pce.70754)
Supplement: Supplementary file 2 [file PCE-49-8400-s002.pdf]

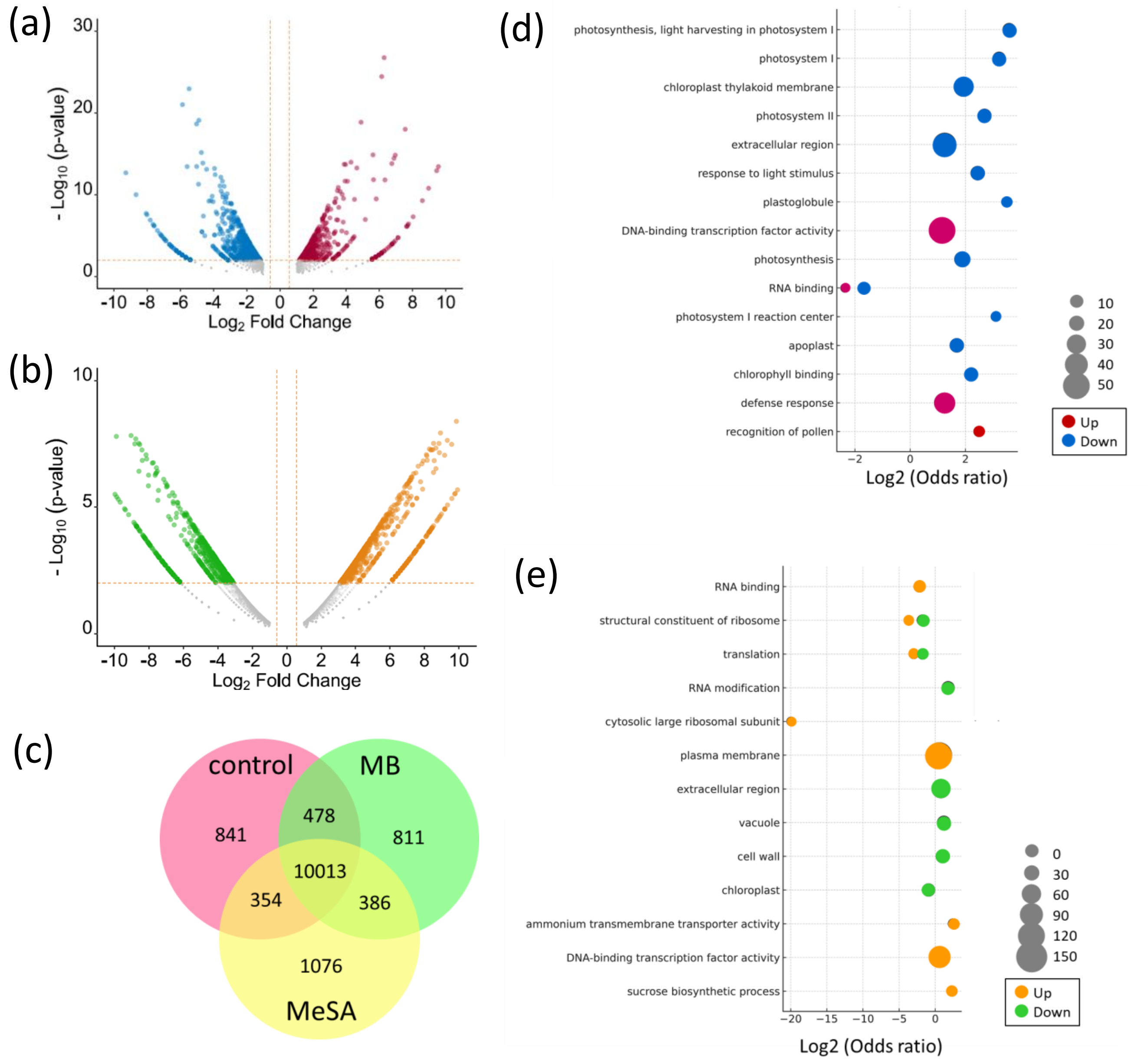

Supporting Figure S5

Comparative transcriptome analysis of tomato leaves treated with MB or MeSA.

(a) Volcano plot showing differentially expressed genes (DEGs) between control and MB-treated (1.35 μg/cm³) leaves. (b) Volcano plot showing DEGs between control and MeSA-treated (1.35 μg/cm³) leaves. Significantly upregulated and downregulated genes ($|log_2FC| \geq 1$, FDR < 0.05) are shown in red and blue (a) or orange and green (b), respectively. (c) Venn diagram illustrating the overlap and specificity of differentially expressed tomato genes induced by MB and MeSA relative to the control treatment. (d) Gene Ontology (GO) enrichment analysis of MB-up and down-regulated genes. Top 15 enriched GO terms (FDR < 0.05) are shown as a bubble plot; bubble size represents gene number, and color denotes $-log_{10}$(FDR). (e) GO enrichment analysis of MeSA-up and down-regulated genes. Top 13 enriched GO terms (FDR < 0.05) are shown as a bubble plot; bubble size represents gene number, and color denotes $-log_{10}$(FDR).
