# Supplementary material for: Methyl Benzoate Acts as a Dusk‐Associated Airborne Cue Activating NPR1‐Dependent Defence Signalling
Source: Plant Cell Environ. 2026 Jul 14;49(11):8400–3. doi: 10.1111/pce.70754 (PMC13646652; doi:10.1111/pce.70754)
Supplement: Supplementary file 3 [file PCE-49-8400-s004.pdf]

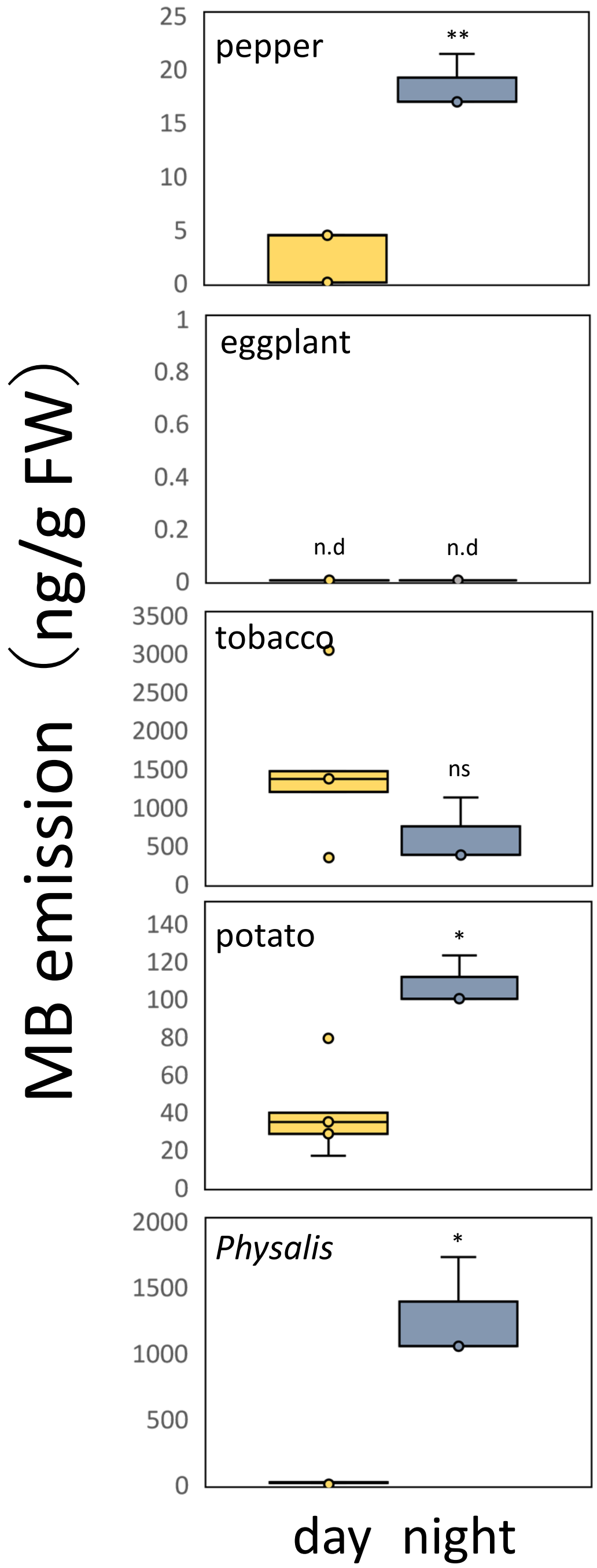

Supporting Figure S2

Day-night comparison of MB emission among *Solanaceae* species.

Volatile emissions were collected using SPME from intact leaves of five *Solanaceae* species—~~tomato~~ pepper (*Capsicum annuum*), eggplant (*Solanum melongena*), tobacco (Nicotiana sylvestris), potato (*Solanum tuberosum*), ~~tobacco (*Nicotiana sylvestris*)~~ and *Physalis peruviana*~~, and eggplant (*Solanum melongena*)~~—during daytime (10:00–17:00) and nighttime (18:00–08:00) under a 14~~2~~ h light/10~~2~~ h dark photoperiod. In all species except eggplant and tobacco, MB emission was higher at night than during the day. The differences were statistically significant in pepper (*p* < 0.01), potato ~~tomato~~ and *Physalis* (*P* < 0.05)~~, while pepper and potato showed the same trend without statistical significance (P > 0.05)~~. MB was not detected in eggplant. Bars represent mean ± SD (n = 5). Asterisks indicate significant differences between day and night periods (*: *P* < 0.05, **: *P* < 0.01, ns: not significant, t-test). n.d, not detected.
