# Supplementary material for: Methyl Benzoate Acts as a Dusk‐Associated Airborne Cue Activating NPR1‐Dependent Defence Signalling
Source: Plant Cell Environ. 2026 Jul 14;49(11):8400–3. doi: 10.1111/pce.70754 (PMC13646652; doi:10.1111/pce.70754)
Supplement: Supplementary file 4 [file PCE-49-8400-s005.pdf]

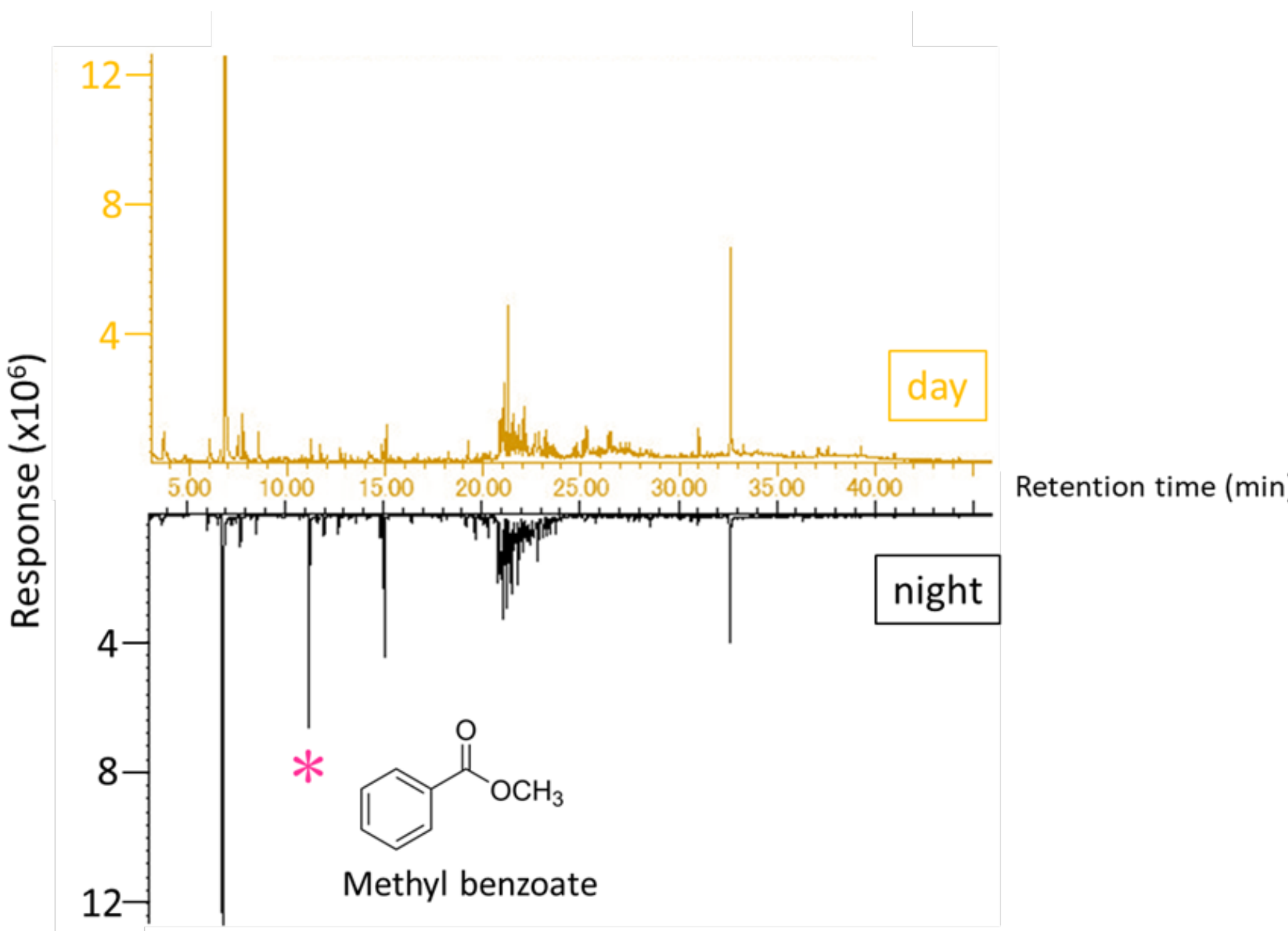

Supporting Figure S1

Day-night comparison of MB emission in tomato leaves.

Volatiles were collected by SPME from intact tomato plants during daytime (10:00–17:00) and nighttime (18:00–08:00) under a 14 h light/10 h dark photoperiod. Representative GC–MS chromatograms show a distinct MB peak (retention time 11.2 min; inset: mass spectrum).
