# Supplementary material for: Methyl Benzoate Acts as a Dusk‐Associated Airborne Cue Activating NPR1‐Dependent Defence Signalling
Source: Plant Cell Environ. 2026 Jul 14;49(11):8400–3. doi: 10.1111/pce.70754 (PMC13646652; doi:10.1111/pce.70754)
Supplement: Supplementary file 5 [file PCE-49-8400-s006.pdf]

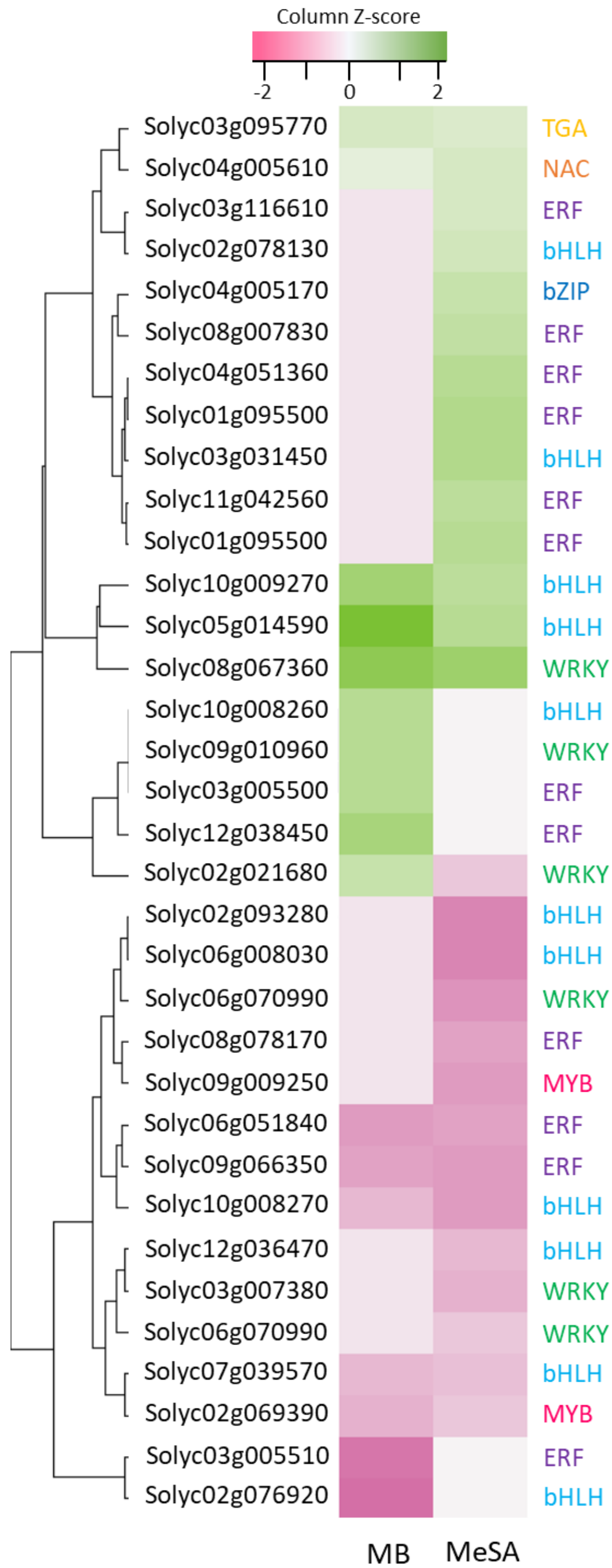

Supporting Figure S6

Differential expression and validation of transcription factors induced by MB and MeSA.

Heatmap showing transcription factor genes differentially expressed in tomato leaves treated with MB or MeSA. Only transcription factor genes with |fold change| ≥ 3 and p-value < 0.05 were included. Color intensity indicates Z-score normalized expression values derived from RNA-seq data.
