# Supplementary material for: Methyl Benzoate Acts as a Dusk‐Associated Airborne Cue Activating NPR1‐Dependent Defence Signalling
Source: Plant Cell Environ. 2026 Jul 14;49(11):8400–3. doi: 10.1111/pce.70754 (PMC13646652; doi:10.1111/pce.70754)
Supplement: Supplementary file 6 [file PCE-49-8400-s007.pdf]

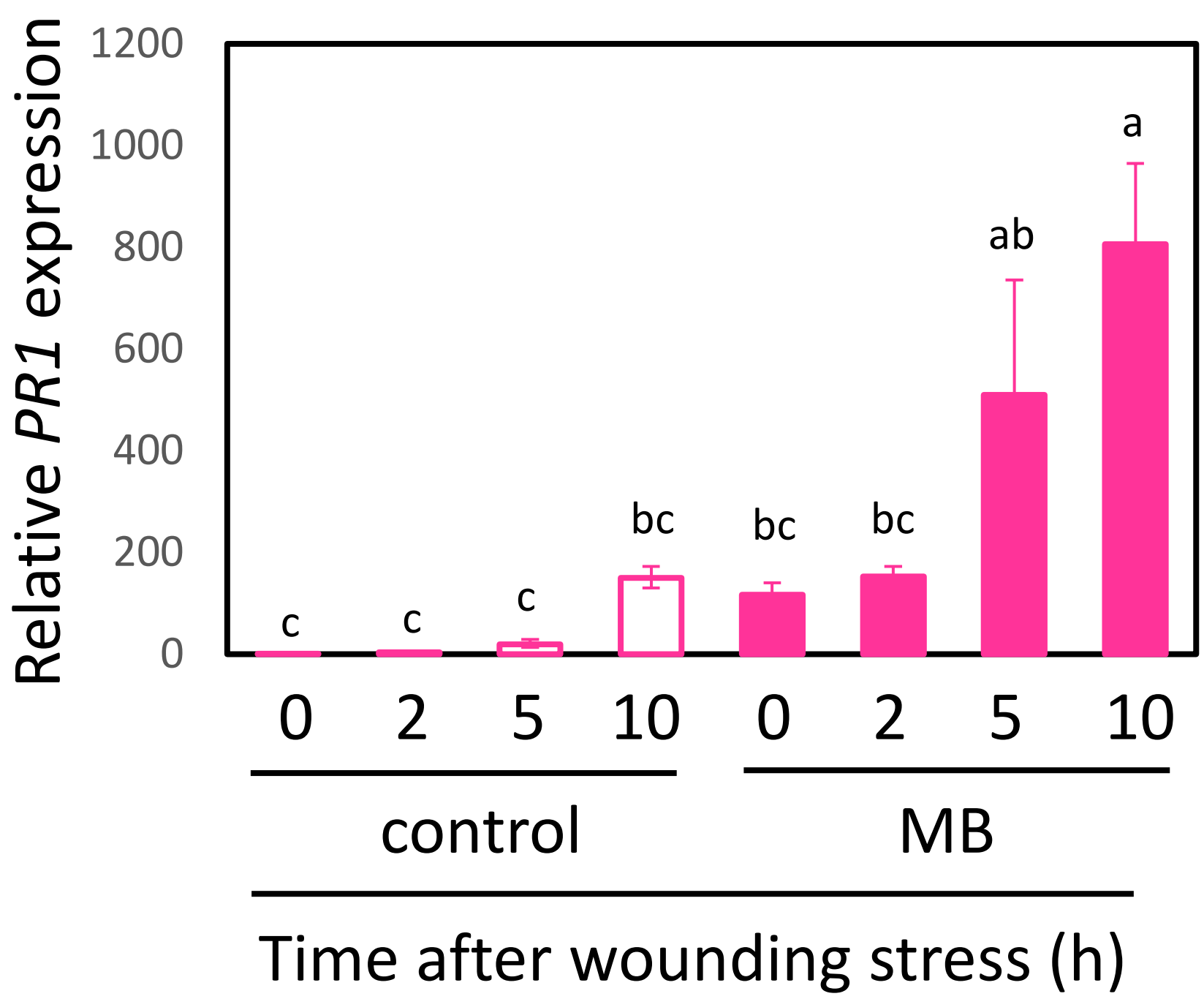


**Supporting Figure S4**

**Priming effect of MB on wound-induced *PR1* expression in tomato leaves.**

Tomato plants were pre-exposed to MB (1.35 μg/cm$^3$) or control air for 24 h and subsequently subjected to mechanical wounding by making shallow incisions on leaf blades using scissors. Relative *PR1* transcript levels were quantified by qRT-PCR at 0, 2, 5, and 10 h after wounding. MB-treated plants exhibited a pronounced priming effect, characterized by earlier and stronger induction of *PR1* expression compared with control plants. Data represent means ± SD (n = 3). Different letters denote significant differences among treatments at each time point (Tukey's HSD test, $P < 0.05$).
