# Supplementary material for: Methyl Benzoate Acts as a Dusk‐Associated Airborne Cue Activating NPR1‐Dependent Defence Signalling
Source: Plant Cell Environ. 2026 Jul 14;49(11):8400–3. doi: 10.1111/pce.70754 (PMC13646652; doi:10.1111/pce.70754)
Supplement: Supplementary file 7 [file PCE-49-8400-s003.docx]

**Supplemental Materials and Methods**

**Plant materials and growth conditions**

Tomato (*Solanum lycopersicum* cv. ‘Micro-Tom’), basil (*Ocimum basilicum* var. minimum), pepper (*Capsicum annuum*), potato (*Solanum tuberosum*), *Physalis peruviana*, eggplant (*Solanum melongena*), tobacco (*Nicotiana sylvestris*), and Arabidopsis (Columbia ecotype) were used in this study. Arabidopsis mutant lines of *npr1-1* (Col background) and *sid2-2* (Col background) were used in this study. All plants were grown in soil consisting of a 1:1 ratio of Metro-Mix (Sun Gro Horticulture, Agawam, MA, USA) to vermiculite within a controlled environmental chamber at 23°C under a 12-h/12-h light/dark photoperiod.

**MB and MeSA treatment**

Three-week-old tomato, pepper, eggplant, and four-week-old Arabidopsis plants grown in 5-cm-diameter pots were placed inside a plant box (7 cm length × 7 cm width × 10 cm height); cotton swabs soaked with MB or MeSA were attached to the bottom of the other plant box, and the box was closed as shown previously in Yoshida et al. (2024). After 4, 8, and 24 h of exposure, plants were removed from the box, their leaves were sampled, quickly frozen in liquid nitrogen, and stored at -80 °C until RNA extraction.

**Quantitative polymerase chain reaction (qPCR) analysis**

Total RNA was isolated from tomato, pepper, eggplant, and Arabidopsis leaves using TRIzol reagent (Invitrogen, Carlsbad, CA, USA). cDNA was synthesized using ReverTra Ace (Toyobo, Osaka, Japan), in accordance with the manufacturer’s instructions. qPCR was performed using the Eco Real-Time PCR System (Illumina, San Diego, CA, USA) using the KAPA SYBR FAST qPCR kit (Sigma-Aldrich, St. Louis, MO, USA). The qPCR cycling protocol consisted of 40 cycles of 95°C for 5 s and 60°C for 30 s. Primer sequences used in this analysis are listed in Table S1. Expression levels for each target gene were normalized to those of the *SlACT* (for tomato), *Ca18S* (for pepper), *SmACT1* (for eggplant), and *ACTIN2* (for Arabidopsis) genes. The replication size was n = 3 for all experiments.

**Volatile collection and GC–MS analysis**

Volatiles emitted from detached tomato leaves were analyzed using a solid-phase microextraction (SPME)–gas chromatography–mass spectrometry (GC–MS) method. A single fully expanded leaf was detached and immediately placed in a 20 mL glass vial sealed with a Teflon-lined septum. After equilibration for 10 min at room temperature (25 °C), a 65 µm PDMS/DVB SPME fiber (Supelco, USA) was inserted into the headspace for volatile collection.

To assess temporal MB emission dynamics, volatile sampling was performed across a full diel cycle under a 14-h photoperiod using SPME-GC–MS. Samples were collected at designated Zeitgeber times (ZT0, ZT4, ZT8, ZT12, ZT16, and ZT20), with each sampling period consisting of 4 h volatile collection prior to analysis. For representative day/night chromatogram comparisons (Supporting Figure S1), SPME sampling was conducted separately during daytime (10:00–17:00) and nighttime (18:00–08:00). During each period, the fiber was continuously exposed within the sealed vial to allow cumulative adsorption of volatiles emitted throughout the respective interval.

Volatile analysis was performed using a GC–MS system (Agilent 7890B GC coupled to a 5977A MSD, Agilent Technologies, USA) equipped with an HP-5MS capillary column (30 m × 0.25 mm i.d., 0.25 µm film thickness). The injector temperature was set to 250 °C, and the SPME fiber was desorbed for 10 min in splitless mode. The oven program was as follows: initial 40 °C (3 min hold), increased to 220 °C at 5 °C min⁻¹, then held for 6 min. Hydrogen served as the carrier gas at 1.0 mL min⁻¹. The MS was operated in electron-impact mode (70 eV) with the ion source at 230 °C and the quadrupole at 150 °C. Mass spectra were recorded from m/z 40–400.

Compounds were identified by comparing retention times and mass spectra with those of authentic standards (Sigma-Aldrich, USA) and the NIST14 library. MB and MeSA were confirmed using authentic reference compounds. Peak areas were normalized to an internal standard (2-octanol, 100 ng µL⁻¹). All analyses were performed using at least three independent biological replicates.

**RNA-seq library preparation and exploratory transcriptome analysis**

For transcriptome profiling, total RNA from MB-, MeSA-, and control-treated tomato leaves (8 h treatment) was submitted to a commercial sequencing service (Rhelixa, Tokyo, Japan).

One representative sample per treatment was used for RNA-seq analysis. Library preparation and sequencing were performed on the Illumina NovaSeq X Plus platform, generating 150 bp paired-end reads. Clean reads were processed and mapped to the tomato reference genome (SL4.0) using HISAT2. Because RNA-seq was conducted with a single biological sample per treatment, this analysis was designed to provide an initial overview of MB- and MeSA-responsive transcriptional changes and to identify candidate genes for subsequent validation. Differentially expressed genes (DEGs) were identified using edgeR with |log₂FC| ≥ 1.0, focusing on qualitative expression trends rather than statistical validation. Gene ontology (GO) enrichment and transcription factor family classification were performed using goatools and PlantTFDB v5.0, respectively.

The RNA-seq dataset was intended as an exploratory transcriptome analysis to identify MB- and MeSA-responsive candidate genes for subsequent qRT–PCR validation.

**Statistical analysis**

All data are presented as mean ± standard deviation (SD) from at least three independent biological replicates. Statistical significance among treatments was determined using a one-way ANOVA followed by Tukey’s multiple-comparison test (p < 0.05).

Use of artificial intelligence (AI) tools

ChatGPT (OpenAI; large language model) was used to assist with language refinement, text editing, and improving manuscript clarity during preparation and revision. All scientific interpretations, experimental design, data analysis, and final editorial decisions were made solely by the authors.

**References**

Yoshida, R., Taguchi, S., Wakita, C., Serikawa, S., and H. Miyaji. 2024. Companion basil plants prime the tomato wound response through volatile signaling in a mixed planting system. *Plant Cell Reports*, 43: 200.
