# Supplementary material for: Methyl Benzoate Acts as a Dusk‐Associated Airborne Cue Activating NPR1‐Dependent Defence Signalling
Source: Plant Cell Environ. 2026 Jul 14;49(11):8400–3. doi: 10.1111/pce.70754 (PMC13646652; doi:10.1111/pce.70754)
Supplement: Supplementary file 8 [file PCE-49-8400-s008.docx]

Supplementary Table

Table S1. Information on primers and genes used in this study.

| Genes name | Forward primer | Reverse primer |
| --- | --- | --- |
| *SlPR1* | TGTCCCAGAGGCCAGACTAT | TTGCAAGAAATGAACCACCA |
| *CaPR1* | CAGGATGCAACACTCTGGTGG | ACCCATCCCAAGGTTCAACT |
| *SmPR1* | GTGGGTCGATGAGAAGCAAT | TACGCCACACCACCTGAGTA |
| *AtPR1* | TTCTTCCCTCGAAAGCTCAA | AAGGCCCACCAGAGTGTATG |
| *AtPR2* | TCCGGTACATCAACGTTGGA | AAGGGAGATTGCTTGCTTGC |
| *AtPR5* | CTTGCCCCGACATGCTTAAG | CGTTTCGTCGTCATAAGCGT |
| *Ca18S* | AAACGGCTACCACATCCAAG | ACCCATCCCAAGGTTCAACT |
| *SmACT* | TGGTCGGAATGGGACAGAAG | CTCAGTCAGGAGAACAGGGT |
| *SlACT* | AGACGCCTATGTGCGGAGATG | GAGGACAGGATGCTCCTCAG |
| *ACTIN2* | AGTGGTCGTACAACCGGTATTGT | GATGGCATGAGGAAGAGAGAAAC |
